# Editorial: The immune escape mechanism and novel immunotherapeutic strategies of leukemia

**DOI:** 10.3389/fimmu.2023.1188582

**Published:** 2023-04-04

**Authors:** Kang Yu, Jialing Zhang, Qianping Li, Shenghui Zhang

**Affiliations:** ^1^ Department of Hematology, The First Affiliated Hospital of Wenzhou Medical University, Wenzhou, Zhejiang, China; ^2^ Institute of Hematology, Wenzhou Medical University, Wenzhou, Zhejiang, China; ^3^ Wenzhou Key Laboratory of Hematology, Wenzhou, Zhejiang, China; ^4^ Laboratory Animal Center, The First Affiliated Hospital of Wenzhou Medical University, Wenzhou, Zhejiang, China

**Keywords:** leukemia, immune escape, immunotherapeutic strategies, regulatory T cells, chimeric antigen receptor T-cell therapy

Increasing evidence unravels that the immune cells within the bone marrow (BM) microenvironment play important roles in the occurrence and development of leukemia (DePasquale et al.; Wurzer et al.) (1). The clinical application of immune checkpoint inhibitors and chimeric antigen receptor (CAR) T-cell therapy has achieved considerable success in treating hematologic malignancies (Aru et al.; Guo et al.; Tomasik et al.; Yang et al.; Hu et al.), but limitations still remain. Therefore, clarifying the interaction of immune cells and leukemia cells and further understanding the mechanisms of immune escape of leukemia cells are the prerequisites for the development of novel immunotherapy strategies.

In this topic, Atene et al. found that chronic lymphocytic leukemia (CLL) cells expressed an active form of indoleamine 2, 3-dioxygenase 1 (IDO1) enzyme and interferon (IFN)-γ secreted from microenvironmental stimuli induced IDO1 expression *via* Jak/STAT1 pathway, and thereby promoting the conversion of tryptophan (Trp) into L-kynurenine (Kyn). Kyn upregulated the expression of myeloid leukemia cell differentiation protein (MCL1) by aryl hydrocarbon receptor (AHR) to promote CLL cell survival. In general, their data identified IDO1/Kyn/AHR signaling as a novel therapeutic target for CLL. Michelis et al. also found that serum alpha-2-macroglobulin (A2M) levels were significantly elevated in CLL patients, possibly due to A2M production by malignant B-lymphocytes. These excess A2M production could upregulate the IgG-hexamerization, resulting in chronic complement activation. Therefore, serum A2M levels were correlated with the disease severity and restraining its overproduction may improve complement activity and immunotherapy outcomes in CLL. TCF1 and its partner gene BCL11B are crucial for sustaining T cell commitment and proliferation, especially maintaining the stem-like properties of CD8^+^ T cells. Liang et al. found that TCF1 and BCL11B were downregulated in CLL patients, especially in CD8^+^ T cells, and significantly correlated with poor time-to-first treatment and overall survival as well as short restricted survival time. More importantly, the combination of TCF1 and BCL11B assessed prognosis more accurately than either alone. In addition, reduced expression of TCF1 and BCL11B, which implied T cell immune dysfunction, was an independent risk factor for rapid disease progression, consistent with high-risk indicators including unmutated IGHV, TP53 changes, and advanced disease.

Grazioli et al. found that functional CD11b^+^Gr-1^+^ myeloid-derived suppressor cells (MDSCs) accumulated in the Notch3-transgenic murine model of T-ALL and aberrant CD4^+^CD8^+^ (DP) T cells from these mice could induce the expansion of MDSCs *in vitro*, as well as in NSG hosts. The MDSC induction was IL-6-dependent and induced MDSCs conversely sustained the expansion and proliferation of DP T-ALL cells. These results clarified a novel role of Notch-dergulated T cells in modifying MDSCs in the T-ALL environment. Jimenez-Morales et al. summarized recent findings on ALL studies and concluded that leukemic cells could avoid immune surveillance through a variety of immunosuppressive mechanisms, and the resulting immune evasioncan in turn boost their proliferation and invasion. How to resist the leukemic cell strategies to deactivate immune cells and favor an immunosuppressive tumor microenvironment (TME) to acquire apoptosis resistance has been applied broadly to develop personalized immunotherapy for ALL. For example, the infusion of co-stimulatory adapted CAR-T cells or neoepitope-specific ALL cells to increase cytotoxic T cell or MHC response is a current option for ALL treatment.

CD4^+^ regulatory T cells (Tregs) are considerably enriched in the BM compared to other secondary lymphoid organs and are critically involved in the establishment of an immune privileged niche to maintain hematopoietic stem cell (HSC) quiescence and to protect HSC integrity. In leukemia, increased Tregs frequency has been recognized as a major immune-regulatory mechanism. Since the cure of leukemia means the elimination of leukemia stem cells (LSCs), it is particularly important to understand these immune-regulatory processes for the development of future treatments of leukemia (Riether). In CLL mouse models, a specific CD44^lo^CD25^lo^ Treg subpopulation was identified and characterized, which was activated to induce an immunosuppressive microenvironment for support of leukemia survival and proliferation. Tregs depletion could trigger the expansion of new anti-leukemic cytotoxic T cell clones to eradicate leukemia. And inhibition of Treg activation with an inhibitor of MALT1 also provided a therapeutic benefit (Goral et al.). Mast cells as immune cells synthesize and store a substantial number of proteases in their secretory granules, including tryptase. Alanazi et al. found that tryptase was also seen in the nuclear compartments of human mast cell line HMC-1. Treatment with cytotoxic agents led to histone 3 cleavage and reduced the levels of several epigentic histone marks, including H3 lysine-4-monomethyl (H3K4me1), H3K9me2, H3 serine-10 phosphorylation (H3S10p), and H2B lysine 16-acetylation (H2BK16ac), in HMC-1 cells. Tryptase inhibition reversed the effects of cytotoxic agents-induced cell death on these epigenetic markers, indicating that it has a profound influence on histone processing. TP53 gene mutations in AML are strongly enriched in complex karyotypes, and are associated with poor prognosis, high tumor mutation load and tumor-infiltrating immune cells, which can be used as biomarkers to predict the immune response to AML (Wen et al.). Myeloid cells as resident immune cells extensively infiltrate the leukemic microenvironment, mainly including populations of neutrophils, macrophages, and MDSCs. These infiltrating cells have been found to be associated with the clinical outcome of the AML patients, and can exert dichotomous functions based on the polarization status of each cell. N2 TANs, M2 TAM and MDSCs show strong immunosuppressive activities that contribute to leukemic progression, acting as a “Yin” role. N1 TANs and M1 TAM stimulate antitumor immune responses, acting as a “Yang” role. Unraveling characterization of the BM immune microenvironment can indicate relevant therapeutic targets and subsequent biomarkers for patients (Magalhaes-Gama et al.).

Although CAR-T cell therapy has revolutionized the treatment of hematological malignancies and achieved a remarkable remission rate, the high recurrence of leukemia after CAR-T cell therapy remains an obstacle to overcome. The value of consolidative transplantation following CAR-T cell-mediated remission is still controversial. Xu et al. conducted a retrospective study and found that patients with R/R B-ALL achieving remission following CD19 CAR-T therapy underwent consolidative unrelated cord blood transplantation (UCBT), conducing to abetter median event-free survival and relapse-free survival (RFS), but without a superior overall survival. And patients with the occurrence of acute graft-versus-host disease (aGVHD) after UCBT had a longer RFS.

The presence of minimal residual disease (MRD) is a well-recognized risk factor for poor prognosis in ALL patients. To investigate the role of CAR-T cell therapy in ALL patients with persistent/recurrent MRD, Hu et al. conducted a respective study and found that 90.7% of these patients achieved MRD negativity after CAR-T cell infusion. Patients who received CAR-T cell therapy had a higher 3-year RFS than those who received chemotherapy bridging to allogeneic HSC transplantation and those who received intensified chemotherapy, suggesting that CAR-T cell therapy can effectively and safely eliminate MRD and significantly improve survival in ALL patients with a suboptimal MRD response. Han et al. reported that a patient with relapsed/refractory Ph^+^ B-ALL received allogeneic HSC infusion to support hematopoiesis after CAR-T cell therapy, and finally, HSC was successfully implanted, suggesting that CAR-T cell therapy can not only induce disease response, but also directly serve as a preconditioning regimen for HSC implantation.

In summary, although great advances have been made in the immune escape mechanism of leukemia in recent years, the roles of some immune cells in the TME have not yet been identified. Immunotherapy provides the possibility of long-term treatment with more specificity and less toxicity for leukemia (2, 3). Increased attention to new immunotherapy strategies and further clarification of the AML pathophysiology have resulted in a better understanding of how TME plays pivotal roles in impeding therapeutic efficacy and exerting toxicity.

## Author contributions

KY, JZ, and QL drafted the manuscript. KY and SZ contributed to the concept, design, and critical revision of the manuscript.
